# Predictive value of CT-based and AI-reconstructed 3D-TAPSE in patients undergoing transcatheter tricuspid valve repair

**DOI:** 10.3389/fcvm.2024.1463978

**Published:** 2025-01-14

**Authors:** Johannes Kirchner, Muhammed Gerçek, Hazem Omran, Kai Peter Friedrichs, Felix Rudolph, Tobias Rossnagel, Misagh Piran, Arseniy Goncharov, Maria Ivannikova, Volker Rudolph, Tanja Katharina Rudolph

**Affiliations:** ^1^ Clinic for General and Interventional Cardiology/Angiology, Herz- und Diabeteszentrum, NRW, Ruhr-Universität Bochum, Medizinische Fakultät OWL (Universität Bielefeld), Bad Oeynhausen, Germany; ^2^ Herz- und Diabeteszentrum, Medizinische Fakultät OWL Universität Bielefeld, Bad Oeynhausen, Germany

**Keywords:** computed tomography, tricuspid regurgitation, transcatheter tricuspis valve repair, TTVI, TAPSE, right ventricular function, transcatheter intervention, tricuspid valve

## Abstract

**Background:**

The tricuspid annular plane systolic excursion (TAPSE) assessed by echocardiography has failed in predicting outcomes in patients with severe tricuspid regurgitation (TR) undergoing transcatheter tricuspid valve intervention (TTVI). Considering the complex shape of the tricuspid annulus and right ventricle, as well as the difficult echocardiographic image acquisition of the right heart, cardiac computed tomography (CT) might be superior for the analysis of the annular excursion. Thus, this study aimed to analyze whether CT-captured TAPSE provides additional value in predicting outcomes after TTVI.

**Methods and results:**

For TTVI procedure planning, 75 patients (mean age, 77 ± 8 years; 61% female) with severe TR underwent full cardiac cycle CT. Septal, lateral, anterior, and posterior TAPSE, as well as TAPSE- volume, were analyzed. Indexed anterior and posterior (iTAPSE) and TAPSE volume were reduced in patients with right ventricular ejection fraction <45%. At 1 year after TTVI (mean follow-up, 193 ± 146days), the combined endpoint of death and rehospitalization occurred in significantly fewer patients with posterior iTAPSE >4.5 mm/m^2^ (17.2% vs. 63.6%; HR 0.225, CI 0.087–0.581; *P* < 0.001) and in patients with iTAPSE volume >9 ml/m^2^ (16.4% vs. 57.1%; HR: 0.269 CI 0.105–0.686; *P* = 0.003). Echocardiographic TAPSE correlated best with lateral CT-based TAPSE, although both failed in predicting outcomes after TTVI. In multivariate Cox regression, posterior iTAPSE was found to be a significant predictor of outcome 1 year after TTVI.

**Conclusions:**

Posterior iTAPSE is an independent predictor of cardiovascular outcomes among patients undergoing TTVI. Furthermore, CT-measured TAPSE has incremental value and refines risk stratification for clinical outcomes in patients undergoing TTVI.

## Introduction

Right ventricular (RV) anatomy and function determine the outcome of several diseases (1). Tricuspid annular plane systolic excursion (TAPSE) is an easy, fast, and reproducible parameter for quantitative assessment of RV function in routine clinical practice. Despite carrying prognostic information in general populations and over several diseases (2, 3), echocardiographic estimated 2D-TAPSE alone has failed in predicting outcomes in patients undergoing transcatheter tricuspid valve intervention (TTVI) (4, 5). Considering the asymmetric structure of the tricuspid annulus, 2D-TAPSE might provide insufficient information about the annulus dynamic during the cardiac cycle. Furthermore, Dreyfus et al. (6) showed that 2D analysis is inferior to 3D analysis regarding tricuspid annulus size in patients with severe tricuspid regurgitation (TR).

Full cycle computed tomography (CT) captures the complex anatomy of the RV and its ejection fraction as well as the tricuspid annular plane throughout the cardiac cycle and has become routine for procedural planning of TTVI (7, 8). To automate cumbersome and time-consuming manual post-processing of CT images, artificial intelligence (AI) augmented software has already found its way into daily cardiac CT and MRI analyses (9–11).

In TR patients, impaired RV function expressed in decreased RV ejection fraction <45% is associated with higher rates of hospitalization and death after TTVI (5). To date, no data exist on whether 3D analysis might be superior to 2D-TAPSE in outcome prediction after TTVI (12, 13).

Thus, we sought to analyze 3D-TAPSE using AI-driven CT reconstruction of the right ventricle and the tricuspid annular plane in patients prior to TTVI and to investigate the predictive value of 3D-TAPSE regarding post-procedure hospitalization and mortality in patients undergoing TTVI.

## Methods

### Study population

In this monocentric study, we included 75 patients who underwent either direct transcatheter annuloplasty using Cardioband (Edwards Lifesciences, Irvine, USA), 55 (73%), or transcatheter edge-to-edge repair using TriClip (Abbott Laboratories, Chicago, USA) or Pascal (Edwards Lifesciences, Irvine, USA), 20 (27%), between May 2020 and June 2023. Patients suffered from severe TR and were deemed unsuitable for cardiac surgery by the heart team. The study was approved and overseen by the Local Ethics Committee, and informed consent for retrospective study inclusion was waived (File number: 2022-945).

### Computed tomography

All patients underwent an electrocardiographically gated, contrast-enhanced CT with full cardiac cycle acquisition using a 320-detector row system (Canon Medical Systems, Japan). Contrast administration was tailored to optimize homogenous right heart enhancement. Axial thin slice (0.5 mm) images were reconstructed in 10% increments throughout the cardiac cycle. All scans exhibited sufficient right-sided contrast opacification for image analysis.

CT data sets were analyzed using heart.ai (LARALAB GmbH, Munich, Germany). heart.ai was used to quantify the right heart morphology for each reconstruction phase throughout the cardiac cycle.

The end-systolic and end-diastolic phases were identified as the phases with minimum and maximum RV volumes. For the CT-based anterior, posterior, septal, and lateral TAPSE measurements, the distance between the systolic and diastolic positions of the tricuspid annulus was measured at the respective locations of the annulus. For TAPSE volume, the annulus of the right ventricle was extracted in each phase. Using the least squares regression method, the best-fit plane was computed for each annulus. The annulus points were projected onto their respective best-fit planes. The annular areas A1 and A2 were calculated using the shoelace formula. The orthogonal distance *D* between the center of mass of the annulus (An1) and the best-fit plane of the annulus (An2) was evaluated. This distance quantifies the displacement between the two annuli. The TAPSE volume was estimated as *D* * (A1 + A2)/2.

### Echocardiographic assessment

All patients received transthoracic echocardiograms within 24–48 h prior to the intervention, with assessment following current American Society of Echocardiography (ASE) guidelines (14). Transthoracic echocardiography (TTE)-based TAPSE was measured using M-mode in the apical four-chamber view as the distance between the most apical and basal position of the tricuspid annulus during one cardiac cycle. The severity of TR was graded according to the five-grade scheme proposed by Hahn and Zamorano (15).

Interbeat changes of TAPSE, fractional area change (FAC), RV mid-cavity diameter (RVMID), and left ventricular (LV) ejection fraction (LVEF) were analyzed in transthoracic echocardiography (TTE) using subsequent beats captured in one series. For evaluation of the dependency of TAPSE on body surface area (BSA), TTEs from 30 healthy subjects were analyzed.

### Procedure

Patients received either transcatheter edge-to-edge repair or direct transcatheter annuloplasty. The interventions were performed under general anesthesia with interventional guidance by transesophageal echocardiography (TEE) and fluoroscopy via femoral vein access. Technical success was defined according to TVARC as successful delivery, deployment, and positioning of the device, absence of procedural mortality, and freedom from emergency surgery related to the device (16).

### Follow-up and outcome

Patients were followed up in our outpatient clinic.

The primary endpoint was a composite of death from any cause or rehospitalization for heart failure (HF). Mortality and HF hospitalizations were assessed by reviewing in-hospital data or by contacting treating general physicians after the index procedure.

### Statistical analysis

Statistical analysis was performed using SPSS Statistics 27 provided by IBM. Continuous and normally distributed variables were expressed as mean ± standard deviation. Receiver operating characteristic (ROC) was performed to determine optimal cutoff values for TAPSE for outcome analysis. Kaplan–Meier analysis was performed for clinical outcomes using time to first event. Differences in time-to-event distributions were evaluated using the log-rank test. The fazard ratio (HR) was calculated using Cox regression. Univariate analyses were initially used, and all parameters with *p* < 0.1 were then included in the multivariate analysis. A *p*-value of <0.05 was considered significant.

## Results

### Study population

Of the 75 enrolled patients, 55 patients (73%) received direct transcatheter annuloplasty, and 20 (27%) received edge-to-edge repair using TriClip (Abbott Laboratories, Chicago, USA) or Pascal (Edwards Lifesciences, Irvine, USA).

The mean patient age was 77 ± 8 years, and 61% were female. The mean EuroScore II was 5.4 ± 4.5%, and most patients were severely symptomatic, presenting in New York Heart Association (NYHA) class ≥3 in 88% (66) of cases. A total of 56 patients (75%) had been previously hospitalized for right-sided heart failure and mean N-terminal pro-B-type natriuretic peptide (NT-proBNP) levels at baseline were 2,850 ± 2,393 ng/L. The most common comorbidities included atrial fibrillation (77%) and heart failure with preserved ejection fraction (68%) (Table 1).

**Table 1:** Baseline data.

	All (*n* = 75)	Posterior iTAPSE >4.5 mm/m^2^ (*n* = 64)	Posterior iTAPSE < 4.5 mm/m^2^ (*n* = 11)	*P*-value
Age, years, mean ± SD	77 ± 8	78 ± 6	72 ± 12	0.15
Female, *n* (%)	46 (61)	43 (67)	3 (27)	**0.018**
Body mass index, kg/m^2^, mean ± SD	27 ± 12	28 ± 13	27 ± 7	0.746
EuroScore II, mean ± SD	5.4 ± 4.5	5.3 ± 4.7	5.4 ± 2.9	0.63
Creatine, mg/dl, mean ± SD	1.6 ± 2.5	1.7 ± 2.7	1.4 ± 0.5	0.759
eGFR, ml/min, mean ± SD	52 ± 23	52 ± 23	52 ± 22	0.968
Kidney impaired, *n* (%)	40 (53)	34 (53)	6 (55)	0.749
NT-proBNP, pg/ml, mean ± SD	2,850 ± 2,393	2,627 ± 2,228	4,141 ± 3,003	0.064
Type of procedure				
Direct annuloplasty, *n* (%)	55 (73)	47 (73)	8 (72)	0.61
Edge-to-edge repair, *n* (%)	20 (27)	17 (27)	3 (27)	
Pacemaker device/ICD implanted, *n* (%)	11 (15)	9 (14)	2 (18)	0.112
History of cardiac decompensation in the past year, *n* (%)	56 (75)	47 (73)	9 (82)	0.433
Coronary artery disease, *n* (%)	31 (41)	25 (39)	6 (55)	0.262
Diabetes mellitus, *n* (%)	14 (19)	12 (19)	2 (18)	0.665
History of stroke, *n* (%)	13 (17)	13 (20)	0 (0)	0.194
COPD, *n* (%)	15 (20)	13 (20)	2 (18)	0.99
Atrial fibrillation, *n* (%)	58 (77)	49 (77)	9 (82)	0.515
History of cardiac surgery, *n* (%)	30 (40)	23 (36)	7 (64)	0.104
HFpEF according to ESC HFAPEFF score, *n* (%)	51 (68)	44 (69)	7 (64)	0.73
Mineralocorticoid receptor antagonist, *n* (%)	35 (47)	29 (45)	6 (55)	0.746
ACE inhibitor or ARB, *n* (%)	47 (63)	38 (59)	8 (73)	0.513
Betablocker, *n* (%)	64 (85)	55 (86)	9 (82)	0.66
DOAC, *n* (%)	47 (63)	40 (63)	7 (64)	0.99
Vitamin-K antagonist, *n* (%)	18 (24)	16 (25)	2 (18)	0.99
Dosage of loop furosemide or torasemide (1:2), mg, median (IQR)	40 (20; 60)	30 (20; 40)	40 (20; 60)	0.589
Baseline NYHA functional class, *n* (%)				
II	9 (12)	9 (14)	0 (0)	0.385
III	63 (84)	52 (81)	11 (100)	
IV	3 (4)	3 (5)	0 (0)	

DOAC, direct oral anticoagulant; HFpEF, heart failure with preserved ejection fraction; NYHA, New York Heart Association.

The bold highlights significant *P*-values.

### TTE data

The mean LVEF was 54 ± 9%. The mean TAPSE was 19 ± 6 mm and was reduced (defined as TAPSE as <17 mm) in 27 cases (36%). The mean vena contracta was 13.4 ± 5.2 mm. Tricuspid regurgitation was graded severe in 32 (43%), massive in 19 (25%), and torrential in 24 (32%) patients (Table 2).

**Table 2:** Transthoracic echocardiography data.

	All (*n* = 75)	Posterior iTAPSE >4.5 mm/m^2^ (*n* = 64)	Posterior iTAPSE <4.5 mm/m^2^ (*n* = 11)	*P*-value
LVEF,%, mean ± SD	54 ± 9	55 ± 9	51 ± 8	0.142
LA diameter, mm, mean ± SD	51 ± 9	50 ± 8	52 ± 12	0.494
RA area, mm^2^, mean ± SD	35 ± 11	35 ± 12	34 ± 5	0.864
RV basal diameter, mm, mean ± SD	48 ± 9	47 ± 9	51 ± 9	0.232
RV FAC,%, mean ± SD	46 ± 12	46 ± 12	44 ± 15	0.543
TV Ring, mm, mean ± SD	43 ± 7	43 ± 7	43 ± 4	0.55
TAPSE, mm, mean ± SD	19 ± 6	19 ± 6	16 ±± 5	0.119
Vena contracta, mm, mean ± SD	13.4 ± 5.2	14 ± 5	12 ± 4	0.314
sPAP, mmHg, mean ± SD	38 ± 14	39 13	38 ± 16	0.388
TR Vmax, m/s, mean ± SD	2.99 ± 0.8	3.1 ± 0.74	2.6 ± 0.9	0.088
TR EROA, mm^2^, mean ± SD	73 ± 44	77 ± 46	52 ± 17	**0.003**
TR RVOL, ml, mean ± SD	64 ± 28	67 ± 28	43 ± 14	**<0.001**
TR at baseline, *n* (%)				
III	32 (43)	29 (45)	3 (27)	0.071
IV	19 (25)	13 (20)	6 (55)	
V	24 (32)	22 (34)	2 (18)	

EROA, effective regurgitation orifice area; FAC, fractional area change; LA, left atrium; LVEF, left ventricular ejection fraction; RA, right atrium; RV, right ventricular; RVOL, regurgitation volume; sPAP, systolic pulmonary artery pressure; TR, tricuspid regurgitation; TV, tricuspid valve.

The bold highlights significant *P*-values.

TTE data were analyzed for interbeat changes of TAPSE, FAC, RVMID, and LVEF which is displayed in Supplementary Table S1. Compared to the mean change of TTE-based TAPSE or LVEF, the mean changes of FAC and RVMID were significantly larger. There was no significant difference in relative change of LVEF compared to TAPSE.

To analyze whether TAPSE is dependent on BSA, we analyzed 30 TTEs from healthy subjects without clinical or echocardiographic RV dysfunction. We found a moderate correlation between TAPSE and BSA (Pearson’s correlation *R* = 0.535, *P* = 0.002).

### CT-TAPSE

**Figure 1:**
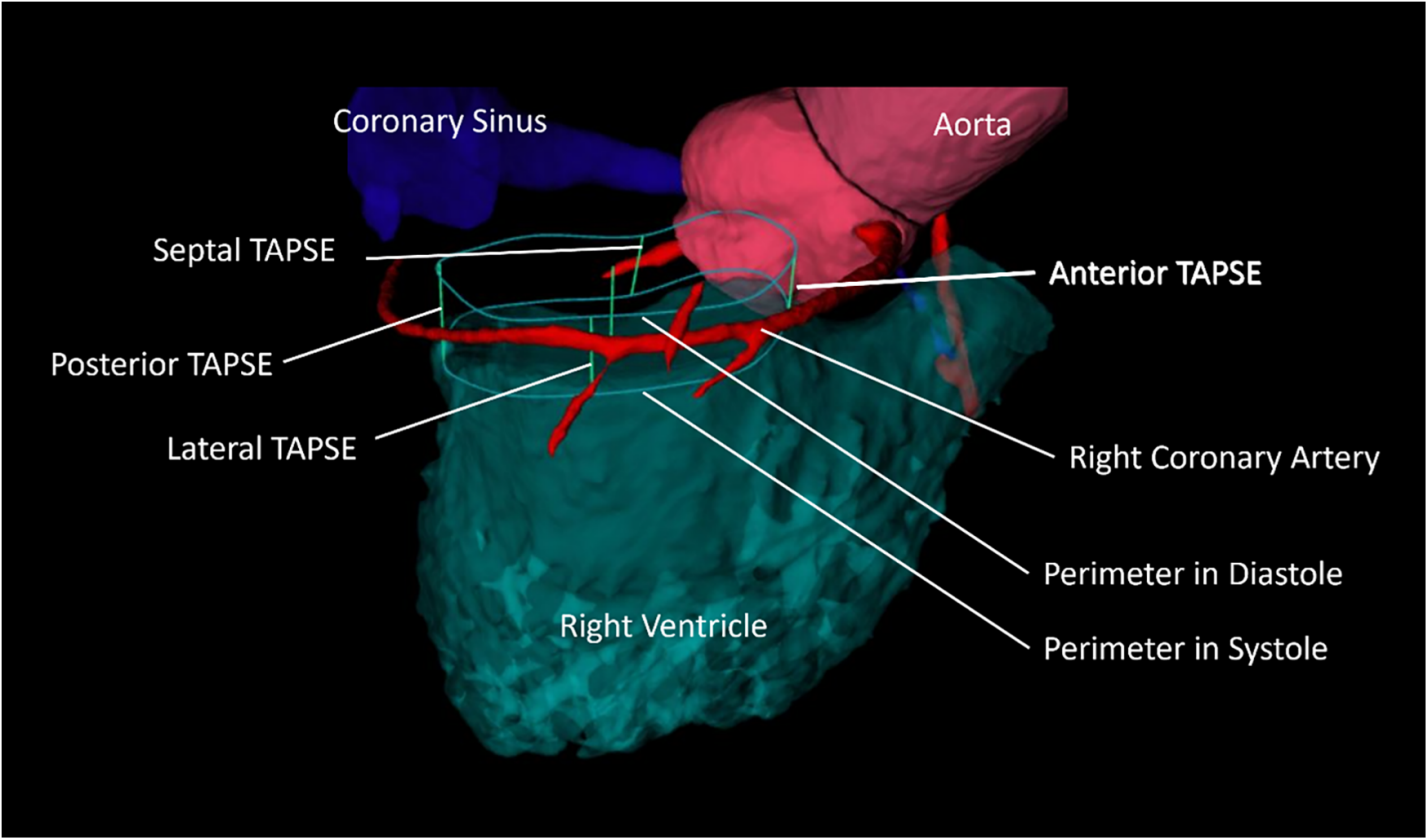
Exemplary illustration of CT-analyzed TAPSE. The illustration depicts the perimeter of the tricuspid valve in diastole and systole and its excursion in anterior, posterior, septal, and lateral locations. The space between the diastolic and systolic position of the perimeter is the “TAPSE volume.”

For further analysis of tricuspid annular excursion, septal, anterior, posterior, and lateral TAPSE was analyzed in CT (Figure 1). The mean septal CT-TAPSE was 15 ± 10 mm, and the lateral CT-TAPSE was 15 ± 11 mm. The anterior TAPSE was 13 ± 6 mm, and the posterior TAPSE was 12 ± 6 mm (Table 3, Supplementary Table S2). There were no significant differences between the TAPSE subgroups in the overall study population. The mean volume of TAPSE between systole and diastole was 13.7 ± 5.6 ml.

**Table 3:** Computed tomography data.

	All (*n* = 75)	Posterior iTAPSE >4.5 mm/m^2^ (*n* = 64)	Posterior iTAPSE <4.5 mm/m^2^ (*n* = 11)	*P*-value
TAPSE septal, mm, mean ± SD	15 ± 10	15 ± 9	11 ± 11	0.121
TAPSE lateral, mm, mean ± SD	15 ± 11	16 ± 11	10 ± 12	0.087
TAPSE anterior, mm, mean ± SD	13 ± 6	13 ± 6	7 ± 5	**0.001**
TAPSE posterior, mm, mean ± SD	12 ± 6	13 ± 5	6 ± 4	**<0.001**
Index TAPSE septal, mm/m^2^, mean ± SD	7.7 ± 4.5	8.1 ± 4.3	5.3 ± 5.2	0.062
Index TAPSE lateral, mm/m^2^, mean ± SD	7.8 ± 5.3	8.2 ± 5.1	4.8 ± 5.3	**0.044**
Index TAPSE anterior, mm/m^2^, mean ± SD	6.6 ± 2.5	7.1 ± 1.9	3.6 ± 2.3	**<0.001**
Index TAPSE posterior, mm/m^2^, mean ± SD	6.5 ± 2.4	7.1 ± 1.9	2.8 ± 1.3	**<0.001**
TAPSE volume, ml, mean ± SD	13.7 ± 5.6	14.4 ± 5.3	9.36 ± 5.38	**<0.002**
Index TAPSE volume, ml/mm^2^, mean ± SD	7.4 ± 3.1	7.8 ± 2.9	4.7 ± 2.4	**0.007**

LV, left ventricular; RV, right ventricular, TAPSE, tricuspid annular plane systolic excursion.

The bold highlights significant *P*-values.

We found a moderate correlation between TTE-measured TAPSE and CT-TAPSE which was best for lateral TAPSE (*R* for Pearson correlation for lateral CT-TAPSE 0.363, *p* < 0.001). Since we found a significant correlation between TTE-TAPSE with BSA, the index values for CT-TAPSE (iTAPSE) were used for further evaluation. Linear regression analysis showed that iTAPSE volume and right ventricular ejection fraction (RVEF) were more closely associated with anterior and posterior iTAPSE than with septal and lateral iTAPSE (Supplementary Table S3).

### Procedural characteristics and outcomes related to CT-TAPSE at 1 year after TTVI

Procedure time and fluoroscopy time were not different in patients with posterior iTAPSE >4.5 mm/m^2^ and <4.5 mm/m^2^ (all *P* > 0.05; Table 4). Compared to patients receiving TEER, patients receiving direct annuloplasty had significantly longer procedure times (187 ± 62 vs. 114 ± 64 min; *P* < 0.001) and fluoroscopy times (52 ± 17 vs. 19 ± 10 min; *P* < 0.01).

**Table 4:** Procedural characteristics and safety profile.

	All (*n* = 75)	Posterior iTAPSE >4.5 mm/m^2^	Posterior iTAPSE <4.5 mm/m^2^	*P*-value
Combined primary outcome, *n* (%)	18 (24)	11 (17)	7 (64)	**0.003**
Rehospitalization for heart failure, *n* (%)	11 (15)	7 (11)	4 (36)	**0**.**049**
Death, *n* (%)	7 (9)	4 (6)	3 (27)	0.06
Procedural characteristics				
Procedure time, minutes, mean ± SD	162 ± 58	160 ± 69	179 ± 47	0.351
Use of contrast medium, ml, mean ± SD	119 ± 63	117 ± 60	82 ± 31	0.663
Fluoroscopy time, minutes, mean ± SD	47 ± 25.9	47.6 ± 26.2	41 ± 26.1	0.681
Type of procedure				
Direct annuloplasty, *n* (%)	55 (73)	47 (73)	8 (72)	0.61
Edge-to-edge repair, *n* (%)	20 (27)	17 (27)	3 (27)	
Technical success, *n* (%)	74 (99)	63 (98)	11 (100)	0.99
Pericardial tamponade, *n* (%)	2 (3)	2 (3)	0 (0)	0.99
Coronary stent implantation for RCA perforation, *n* (%)	1 (1)	0 (0)	1 (9)	0.145
Pericarditis, *n* (%)	2 (3)	2 (3)	0 (0)	0.99
In-hospital death, *n* (%)	1 (1)	1 (2)	0 (0)	0.99
Transient AV blockage °III, *n* (%)	5 (7)	5 (8)	0 (0)	0.99
Periprocedural dialysis, *n* (%)	1 (1)	(0)	1 (9)	0.145
Access associated bleeding requiring transfusion, *n* (%)	1 (1)	1 (2)	0 (0)	0.99

Values are displayed as mean ± SD or frequencies (%). AV, atrioventricular; RCA, right coronary artery.

The bold highlights significant *P*-values.

The mean follow-up was 193 ± 146 days. During the follow-up time, 18 patients reached the primary endpoint, of whom 11 patients were hospitalized for heart failure and 7 patients died (Table 4).

The receiver operator curve showed the largest area under the curve for the primary outcome for posterior iTAPSE (AUC 0.72) and iTAPSE volume (AUC 0.72, Supplementary Figure 2). For outcome prediction, a posterior iTAPSE of 4.5 mm/m^2^ and an iTAPSE volume of 9 ml/m^2^ performed best.

**Figure 2:**
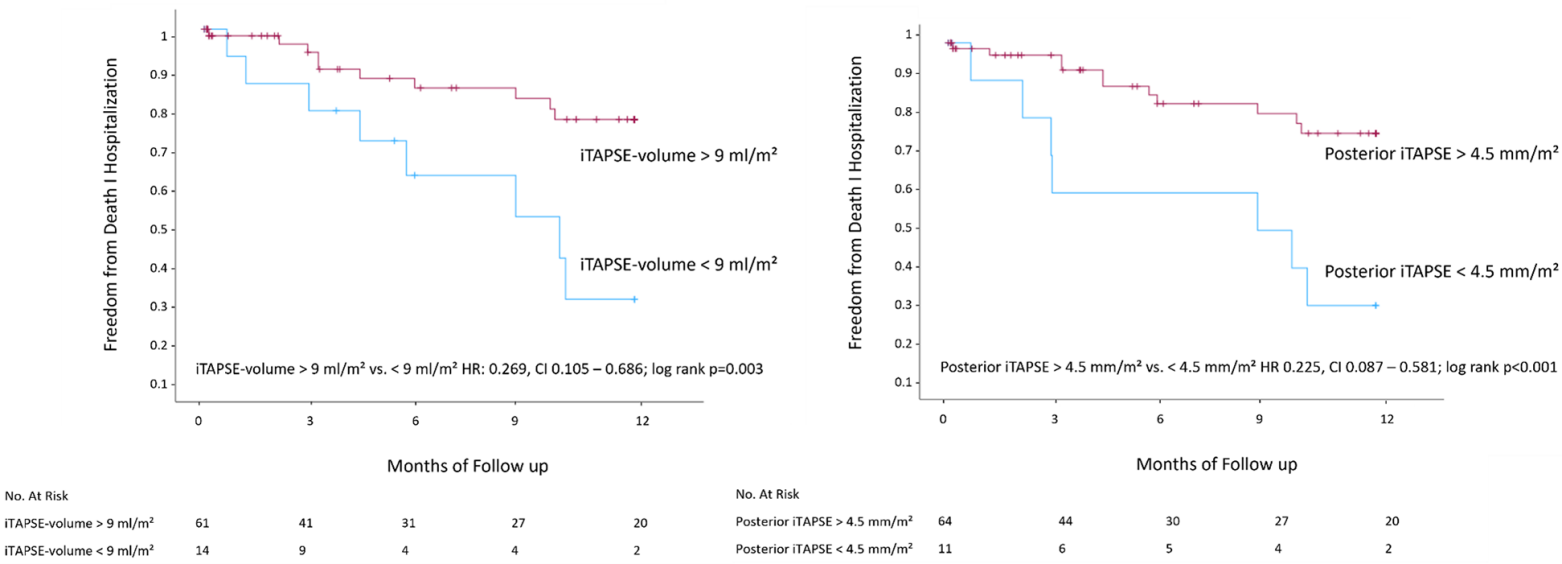
Kaplan–Meier analysis of posterior iTAPSE and iTAPSE volume. CT, computed tomography; i, indexed; TAPSE, tricuspid annular plane systolic excursion.

At 1 year, the estimated combined endpoint was observed significantly less frequently in patients with posterior iTAPSE >4.5 mm/m^2^ (17.2% vs. 63.6%; HR 0.225, CI 0.087–0.581; *P* < 0.001; Figure 2). Furthermore, an iTAPSE volume of >9 ml/m^2^ was also associated with better outcome (16.4% vs. 57.1%; HR: 0.269 CI 0.105–0.686; *P* = 0.003).

Then we divided the cohort into three subgroups. In the first group, patients had a preserved RVEF and preserved posterior iTAPSE (defined as >4.5 mm/m^2^). In the second group, patients had either a preserved posterior iTAPSE or a preserved RVEF, and in the third group, there were patients with reduced TAPSE and reduced RVEF. We found that patients with impaired RVEF and iTAPSE and patients who had either preserved RVEF or iTAPSE had comparable outcomes (Supplementary Figure S2). In this subgroup, eight patients had reduced RVEF but preserved iTAPSE, and six patients had reduced iTAPSE but preserved RVEF.

Univariate analysis showed that echocardiographic-measured LVEF and artificial intelligence-measured CT RVEF, posterior iTAPSE, and TAPSE volume were associated with the primary outcome at 1 year. On multivariate Cox regression, posterior iTAPSE remained a significant predictor for outcomes (*p* = 0.022, Table 5). Both TTE-measured TAPSE (HR 1.013, CI 0.918–1.117; *p* = 0.803) and CT-measured lateral TAPSE (HR 0.919, CI 0.837–1,009; *p* = 0.075) failed in predicting outcomes at 1 year.

**Table 5:** Uni- and multivariate Cox regression.

Value	Hazard ratio (95%CI)	*P*-value for Cox regression
Univariate analyses		
Age	0.932–1.048	0.69
Gender	0.659–4.199	0.281
EuroScore II	0.964–1.176	0.215
Diabetes mellitus	0.393–4.782	0.622
NYHA class	0.158–7.611	0.937
eGFR	0.969–1.016	0.502
TTE LV ejection fraction	0.909–0.992	**0**.**021**
TTE-TAPSE	0.918–1.117	0.803
TTE fractional area change	0.956–1.028	0.626
TR at baseline	0.816–2.35	0.228
TR post procedure	0.538–1.423	0.59
CT-RV ejection fraction	0.893–0.998	**0**.**043**
iCT TAPSE volume	0.711–0.988	**0**.**035**
iCT posterior TAPSE	0.632–0.946	**0**.**013**
Multivariate analyses		
Model 1		
TTE LVEF	0.916–1.012	0.139
CT RVEF	0.904–1.026	0.243
Model 2		
TTE LVEF	0.919–1.001	0.058
iCT posterior TAPSE	0.594–0.961	**0**.**022**
Model 3		
TTE LVEF	0.917–1.001	0.056
iCT TAPSE volume	0.692–1.016	0.072

CT, computed tomography; eGFR, estimated glomerular filtration rate; LV, left ventricular; NYHA, New York Heart Association; RV, right ventricular, TAPSE, tricuspid annulus plane systolic excursion; TR, tricuspid regurgitation; TTE, transthoracic echocardiography.

The bold highlights significant *P*-values.

The prevalence of atrial fibrillation was 77% in our cohort. When analyzing only patients with atrial fibrillation, we found that posterior iTAPSE (HR 0.647 CI 0.475–0.881; *p* = 0.006) but not RVEF (HR 0.955 CI 0.9–1.012; *p* = 0.12), both evaluated as continuous variable, were significantly associated with the combined endpoint.

## Discussion

Here, we present the first study to evaluate AI-reconstructed tricuspid annular excursion in CT scans for patients undergoing TTVI. The main findings of the study are:

1) Echocardiographic-measured TAPSE showed a moderate correlation with CT-measured lateral TAPSE, although both failed in predicting outcomes after TTVI.
2) Longitudinal RV function evaluated as three-dimensional CT-TAPSE depends rather on anterior and posterior annular excursion than on septal or lateral excursion.
3) On multivariate Cox regression, posterior iTAPSE remained a significant predictor with incremental value for the risk of hospitalization and death after TTVI.

To address the complex anatomy of the tricuspid annulus, we separately analyzed annular excursion in septal, anterior, posterior, and lateral positions. After stratification for RVEF, we found posterior and anterior iTAPSE significantly reduced in patients with impaired RVEF whereas septal and lateral iTAPSE were not different in patients with preserved or impaired RVEF.

In autopsies, the anterior and posterior papillary muscle (PM) was found to be larger than the septal PM whereas the septal PM was morphologically more heterogenous and often completely absent (17). Therefore, the anterior and posterior contraction might be decisive for the preservation of RV function in chronic volume overload, and loss of anterior and posterior contraction might ultimately impair RV heart function more than septal and lateral contraction. As lateral CT-TAPSE correlated best with TTE-measured TAPSE, this might explain why TTE-measured TAPSE was shown to be non-predictive in patients undergoing TTVI (18, 19).

Then, the volume of the annular excursion was measured as cylinder-like shape delimited by the diastolic and systolic position of the tricuspid annulus (Figure 1). Anterior and posterior iTAPSE was significantly different whereas septal and lateral iTAPSE was not different in patients with bigger TAPSE volume compared to patients with smaller TAPSE volume. This underlines the hypothesis that anterior and posterior contraction is more decisive for longitudinal RV function than septal and lateral contraction.

Additionally, we analyzed whether CT-TAPSE is associated with better outcomes after TTVI. We found that higher index posterior TAPSE and TAPSE volume were associated with a better outcome at 1 year. Kaplan–Meier analysis revealed significantly better outcomes in patients with posterior iTAPSE >4.5 mm/m^2^ and TAPSE volume >9 ml/^2^. However, the ROC showed that the complex measurement of iTAPSE volume was not superior to the rather straightforward posterior iTAPSE measurement in relation to outcome prediction. Furthermore, TAPSE volume depends on the annulus area and annular excursion. Hence, a relatively small systolic excursion with a large annulus area might create a substantial TAPSE volume whereas a small annulus area with a larger systolic excursion might generate a smaller TAPSE volume. Therefore, posterior TAPSE rather than TAPSE volume might be used in future studies.

RVEF has been shown to be predictive for patients undergoing TTVI (5, 20). In our analysis, combined RVEF and TAPSE analysis showed that the subgroup, being 18.6% of the study population, with either reduced CT-TAPSE or RVEF had comparable outcomes compared to patients with both reduced TAPSE and RVEF. This would redefine outcome prediction for nearly 10% (6 out of 62) of the patients with preserved RVEF and underline that CT-TAPSE has incremental value in TTVI outcome prediction.

In patients with severe tricuspid regurgitation, the prevalence of atrial fibrillation is high. The irregular heart rhythm leads to constantly changing preload conditions of the ventricles and varying stroke volume over time. In healthy individuals, the Frank–Starling mechanism links the length of myocardial fibers and force of contraction allowing for adjustment to changing preload conditions. In heart failure, the Frank–Starling mechanism is impaired, and the right ventricle often fails to adapt to rapidly changing preload conditions (21–23). Whether these changes in preload also extend to the level of changing R–R intervals, as in atrial fibrillation, is unclear. Since FAC correlates with RVEF, we used TTE to detect changes in FAC in two consecutive cardiac cycles and compared the relative differences with changes in TAPSE (24, 25). We found that compared to relative changes in TAPSE, FAC and RVMID mean changes were significantly higher whereas LVEF was not significantly affected. Because of the fair correlation of FAC and RVEF, this might indicate that the mean difference of RVEF is also affected by changing R–R intervals. Furthermore, changes in radial contraction might be more prone to arrhythmia than longitudinal contraction since the mean differences of RVMID were bigger than the mean changes of TAPSE. This is interesting because in MRI-measured RVEF, increased radial and circumferential strain compensates for the loss of longitudinal strain in patients with preserved RVEF (5). Hence, the contraction pattern that distinguishes patients with preserved or reduced RVEF might be mostly affected by arrhythmia. As in our study, cardiac volumetrics with full cycle cardiac CT is usually undertaken in only one cardiac cycle. Therefore, CT measurements might be more susceptible to interbeat variability-induced change of RVEF compared to cardiac MRI. However, to address this question in particular, one might need to analyze interbeat variability of RVEF and RVMID as well as TAPSE with CT over several cardiac cycles which might cause inordinate radiation doses.

Interestingly, in patients with atrial fibrillation, posterior iTAPSE but not RVEF was significantly associated with the primary outcome. This might be in part attributed to a reduced sample size, as only 58 out of 75 patients were included. However, this points out that in patients with arrhythmia mere RVEF might provide insufficient information for RV function.

## Limitations

This is a study from a single center focusing on patients who underwent pre-procedural CT prior to TTVI. Compared to other studies, the study population is small which might introduce selection bias. Due to the lack of data on right ventricular ejection fraction (RVEF) or CT-TAPSE measurements across multiple cardiac cycles, only assumptions can be made regarding the correlation between 2D TTE measurements of fractional area change (FAC) and TTE-TAPSE with 3D changes in CT scans of RVEF and CT-TAPSE. More studies are needed to further evaluate the value of CT-based TAPSE.

Furthermore, it is under debate which cutoff value for RVEF is appropriate in patients with severe tricuspid regurgitation. Often CT RVEF of 50% or more is considered normal, but, as in our study, several studies analyzing patients with severe tricuspid regurgitation defined >45% RVEF as preserved (26, 5, 20).

## Conclusion

Anterior and posterior annular excursion is reduced in patients with right ventricular failure. Posterior iTAPSE is an independent predictor of cardiovascular outcomes among patients undergoing TTVI. Considering the prevalence and interbeat variability in patients with atrial fibrillation, the combination of CT-measured TAPSE and RVEF might refine risk stratification for clinical outcomes in patients undergoing TTVI.

## Data Availability

The raw data supporting the conclusions of this article will be made available by the authors, without undue reservation.
