# Supplementary material for: Predictive value of CT-based and AI-reconstructed 3D-TAPSE in patients undergoing transcatheter tricuspid valve repair
Source: Front Cardiovasc Med. 2025 Jan 14;11:1463978. doi: 10.3389/fcvm.2024.1463978 (PMC11772429; doi:10.3389/fcvm.2024.1463978)
Supplement: Supplementary file 1 [file Datasheet1.pdf]

Supplementary Table 1. Interbeat Variability

	Mean ± SD<br>Or<br>Median (IQR)	P-value compared to Change in TAPSE
Change Frequency, %, mean ± SD	16.3 ± 15.5	0.168
Change TAPSE, %, mean ± SD	7.3 ± 6.5	-
Change FAC, %, mean ± SD	29.8 ± 27.8	<0.001
Change RVMID, %, median (IQR)	35.42 (15.79; 75.64)	<0.001
Change LV ejection fraction, %, mean ± SD	11.3 ± 7.3	0.99

Interbeat changes of TAPSE, fractional area change (FAC), RV mid-cavity diameter (RVMID) and left ventricular (LV) ejection fraction (LV EF) were analyzed in transthoracic echocardiography (TTE) using subsequent beats captured in one series.; TAPSE, tricuspid annulus plane systolic excursion

Supplementary Table 2. Computed tomography data.

	All (n=75)	Posterior iTAPSE > 4.5 mm/m² (n=64)	Posterior iTAPSE < 4.5 mm/m² (n= 11)	P-value
LV end diastolic volume, ml, mean ± SD	124 ± 37	122 ± 32	134 ± 33	0.33
LV end systolic volume, ml, mean ± SD	55 ± 27	53 ± 26	68 ± 27	0.08

LV stroke volume, ml, mean ± SD	69 ± 19	70 ± 19	67 ± 12	0.606
LV ejection fraction, %, mean ± SD	57 ± 11	58 ± 10	50 ± 10	**0.023**
LA Volume diastolic, ml, mean ± SD	157 ± 74	155 ± 67	175 ± 109	0.403
LA Volume systolic, ml, mean ± SD	179 ± 75	176 ± 68	193 ± 110	0.493
RV end diastolic volume, ml, mean ± SD	258 ± 72	254 ± 65	287 ± 102	0.333
RV end systolic volume, ml, mean ± SD	127 ± 48	121 ± 40	167 ± 75	0.094
RV stroke volume, ml, mean ± SD	145 ± 112	149 ± 119	119 ± 35	0.441
RV ejection fraction, %, mean ± SD	52 ± 8	53 ± 7	44 ± 7	**<0.001**
RA volume diastolic, ml, mean ± SD	270 ± 117	268 ± 123	282 ± 67	0.719
RA volume systolic, ml, mean ± SD	305 ± 124	306 ± 130	301 ± 130	0.589
Tricuspid annulus area diastolic, mm^2, mean ± SD	1772 ± 402	1796 ± 352	1633 ± 626	0.417
Tricuspid annulus perimeter diastolic, mm, mean ± SD	150 ± 18	150 ± 14	145 ± 33	0.554
Tricuspid annulus area systolic, mm, mean ± SD	1629 ± 362	1631 ± 322	1619 ± 562	0.923
Tricuspid annulus perimeter systolic, mm, mean ± SD	143 ± 18	143 ± 14	140 ± 32	0.784
TAPSE septal, mm, mean ± SD	15 ± 10	15 ± 9	11 ± 11	0.121
TAPSE lateral, mm, mean ± SD	15 ± 11	16 ± 11	10 ± 12	0.087

TAPSE anterior, mm, mean ± SD	13 ± 6	13 ± 6	7 ± 5	**0.001**
TAPSE posterior, mm, mean ± SD	12 ± 6	13 ± 5	6 ± 4	**<0.001**
Index TAPSE septal, mm/m², mean ± SD	7.7 ± 4.5	8.1 ± 4.3	5.3 ± 5.2	0.062
Index TAPSE lateral, mm/m², mean ± SD	7.8 ± 5.3	8.2 ± 5.1	4.8 ± 5.3	**0.044**
Index TAPSE anterior, mm/m², mean ± SD	6.6 ± 2.5	7.1 ± 1.9	3.6 ± 2.3	**<0.001**
Index TAPSE posterior, mm/m², mean ± SD	6.5 ± 2.4	7.1 ± 1.9	2.8 ± 1.3	**<0.001**
TAPSE-volume, ml, mean ± SD	13.7 ± 5.6	14.4 ± 5.3	9.36 ± 5.38	**<0.002**
Index TAPSE-volume, ml/mm², mean ± SD	7.4 ± 3.1	7.8 ± 2.9	4.7 ± 2.4	**0.007**

LV, Left ventricular; RV, Right ventricular, TAPSE, tricuspid annular plane systolic excursion

Supplementary Table 3. Dependency of RV EF on TAPSE.

	R^2 for RV EF	*Linear regression*	P-Value
Septal iTAPSE	0.087	*-0.543+0.156x*	0.011
Anterior iTAPSE	0.244	*-0.955+0.147x*	<0.001
Posterior iTAPSE	0.372	*-2.464+0.175x*	<0.001
Lateral iTAPSE	0.059	*-0.383+0.154x*	0.038
Volume of iTAPSE	0.145	*0.111+0.142x*	<0.001

iTAPSE, indexed tricuspid annulus plane systolic excursion; RV EF, right ventricular ejection fraction

Supplementary Figure 1. Receiver operator characteristic for posterior iTAPSE (A) and iTAPSE-volume (B)

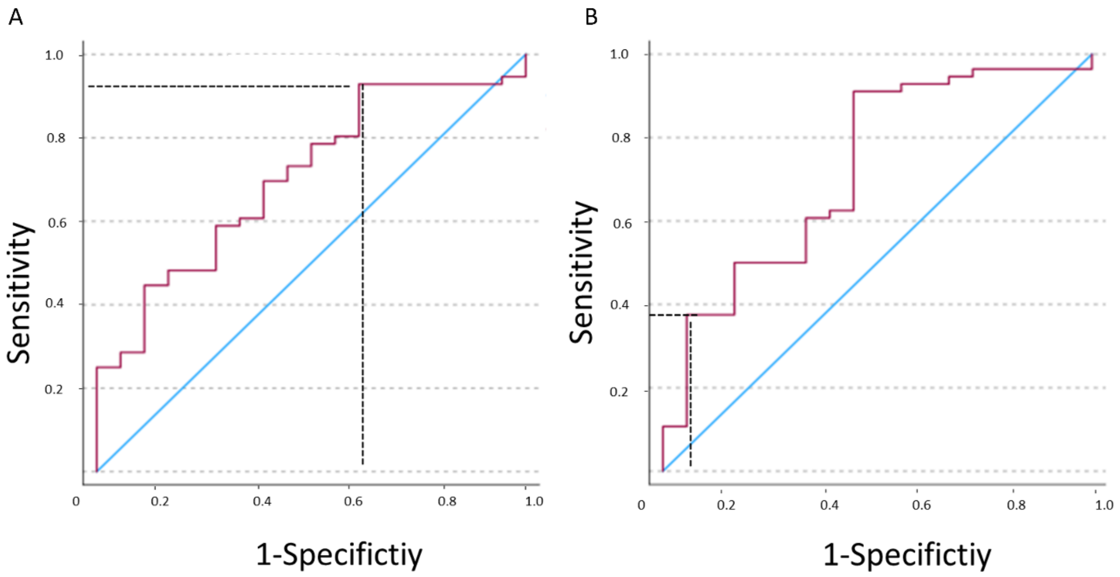

Receiver Operator characteristic for posterior iTAPSE (A) and iTAPSE-volume (B). The dotted lines mark sensitivity and 1-specificity for posterior iTAPSE >4.5mm/m² and iTAPSE-volume >9ml/m². iTAPSE, indexed tricuspid annular plane systolic excursion.

Supplementary Figure 2. Kaplan-Meier-Analysis of posterior iTAPSE and RV EF

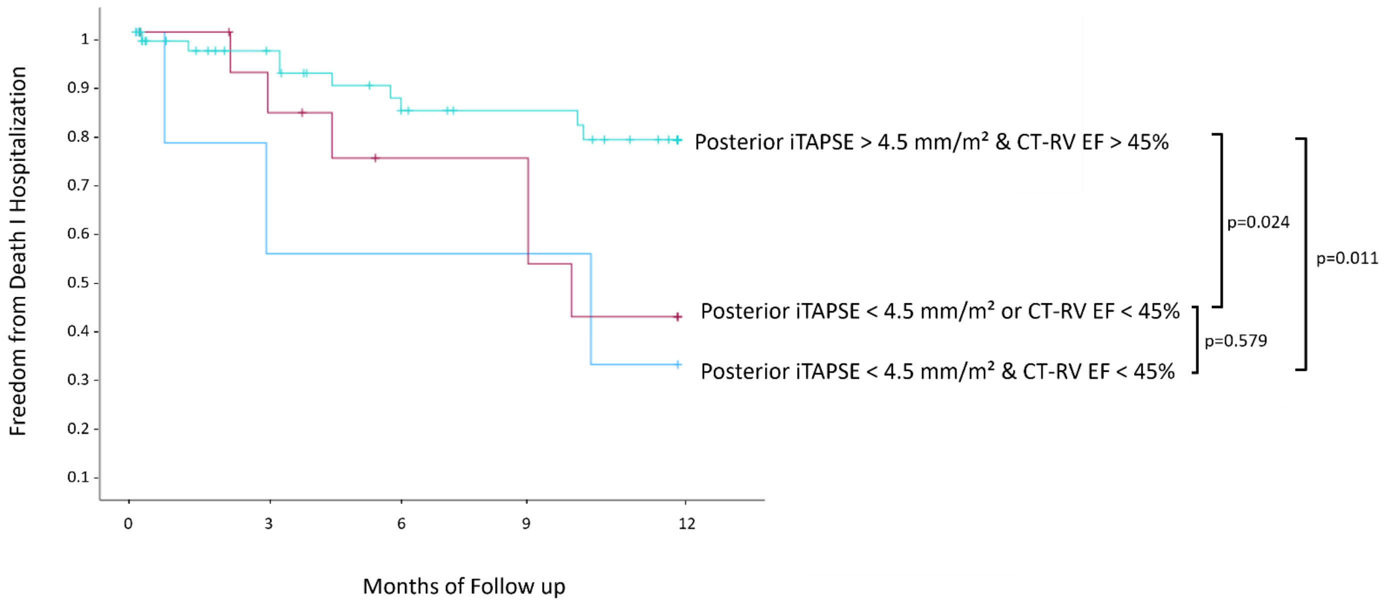

No. At Risk					
Posterior iTAPSE > 4.5 mm/m² & CT-RV EF > 45%	56	38	28	25	18
Posterior iTAPSE < 4.5 mm/m² or CT-RV EF < 45%	14	10	5	4	3
Posterior iTAPSE < 4.5 mm/m² & CT-RV EF < 45%	5	2	2	2	1

CT, computed tomography; i, indexed; RV EF, right ventricular ejection fraction; TAPSE, tricuspid annular plane systolic excursion.
